# Quickly Identifying High-Risk Variables of Ultrasonic Extraction Oil from Multi-Dimensional Risk Variable Patterns and a Comparative Evaluation of Different Extraction Methods on the Quality of *Forsythia suspensa* Seed Oil

**DOI:** 10.3390/molecules24193445

**Published:** 2019-09-23

**Authors:** Liangshan Ming, Hao Huang, Yumao Jiang, Gengjinsheng Cheng, Daoying Zhang, Zhe Li

**Affiliations:** 1 National Engineering Research Center for Modernization of Traditional Chinese Medicine (TCM)-Hakka TCM Resource Branch Center, School of Pharmacy, Gannan Medical University, Ganzhou 341000, China; hwaohuanghao@gmail.com (H.H.); 13683301305@163.com (Y.J.); gjsgmu625@163.com (G.C.); zhangdaoying2004@163.com (D.Z.); 2 Key Laboratory of Preparation of Modern TCM, Ministry of Education, Jiangxi University of TCM, Nanchang 330004, China; 3 Artemisinin Research Center, China Academy of Chinese Medical Sciences, Beijing 100700, China

**Keywords:** *Forsythia suspensa*, ultrasonic extraction, L18-Hunter design, GC-MS analysis, antioxidant activity

## Abstract

Generally, essential oils and components of interest are extracted from plants using organic solvent, distillation, ultrasound and supercritical extraction methods. Ultrasonic extraction (UE) has the advantage of high efficiency, but its process is complicated and it has numerous variables. In this study, an L18-Hunter experimental design was applied for the first time to investigate the practicability of applying UE to *Forsythia suspensa* seed oil. Six potential high-risk variables, including numerical and non-numeric types, were obtained from the risk analysis and their impacts on global yield and antioxidant activity were screened. Furthermore, oils obtained by different extraction processes (i.e., UE, supercritical fluid extraction (SFE), soxhlet extraction (SE) and hydrodistillation extraction (HD)) were analyzed. A comparative study of these oils was characterized and compared by FT-IR, GC-MS and antioxidant activity. The obtained results show that the type of solvent, solvent-to-solid ratio, extraction power and time were the significant variables affecting the extraction yield, whereas antioxidant activity was only affected by the type of solvent. The regression coefficients of the yield and antioxidant activity models were 0.79 and 0.91, and the ANOVA of the models were 0.013 and <0.0001, respectively. Beta-Pinene was the main abundant component in the oils for the UE, SFE, SE and HD methods and the content was about 46%~52.4%. In conclusion, the L18-Hunter design could be used as an effective experimental design method for rapid screening of high-risk variables. Regarding extraction efficiency, chemical composition and biological activity, UE not only offered a robust *Forsythia suspensa* seed oil extraction process, but also provided a time- and cost-effective advantage to the food and pharmaceutical industry when compared to the SFE, SE and HD extraction processes.

## 1. Introduction

*Forsythia suspensa* ((Thunb) vahl (Oleaceae)) is a deciduous shrub plant that is widely distributed in China, Southeast Asia and many European countries [1]. The seeds of this plant exhibit high pharmacological activity and are used widely in the treatment of diseases. Previous studies have demonstrated that *Forsythia suspensa* seed oil is one of the principal bioactive components in the plant, and it has antiviral, antioxidant, antibacterial, anti-inflammatory activities [2,3]. Due to its medicinal values and functionality, it is highly desirable to develop an efficient method for extracting seed oil with a high yield and to determine its activity.

Ultrasonic extraction (UE) has been widely used in the modern plant-based chemistry industry, from laboratory to industrial scale. All the applications reported in the literature showed that UE is a green economical alternative to conventional techniques for food and pharmaceuticals [4,5] because of the decrease of processing time, energy and solvent used. These benefits are mainly determined by the mechanism of UE. In the UE operation, materials are subjected to the effects of fragmentation, erosion, capillarity, detexturation and sonoporation, so that the biological active components are rapidly released from cells and tissues [6]. Currently, it has been successfully applied to the extraction of active ingredients from different parts of plants such as roots, stems, leaves, seeds and fruits [7,8]. In addition to UE, conventional solvent reflux extraction and modern supercritical fluid extraction are also commonly used extraction methods [9,10].

The influencing factors and variables of the extraction process are complex, and its variables are numerous. Therefore, the procedure for screening out high-risk variables from a large number of variables is very complicated. Determining the impact of critical variables on extraction efficiency and establishing mathematical models have become the research hotspots and frontiers of researches [11]. There are many factors affecting extraction efficiency during the UE process. At present, researches are mainly focusing on the effects of extraction power, the amount of solvent used and ultrasonic time on the ultrasonic results [12,13]. Some researchers have also compared the difference in the physiochemical characteristics of various extraction processes. For example, Hu et al. conducted an orthogonal experiment to investigate the effects of ultrasonic power and temperature and the water to material ratio on the extraction of polysaccharides from the Chuanxiong rhizome [14]. In another study by Sicaire et al., a response surface methodology with a three-variable central composite design was used to explore the effect of these three variables on the oil extracted from oleaginous seeds [15]. The physiochemical characteristics, including antioxidant activity, crystallization and melting behavior of *Moringa peregrina* oil extracts, obtained through UE and Soxhlet treatment were determined and compared [13]. These research results have enhanced the understanding of the ultrasonic process and promoted the utilization of UE.

However, it is still important to note that in addition to the above-mentioned influencing variables, other variables, such as solvent parameters and raw material properties, are usually ignored, but these variables are also important and need to be studied deeply. Further analyses have found that researchers are eager to study these variables, which may be discontinuous or non-numeric features. Thus, commonly used statistical analysis methods such as response surface and orthogonal experimental designs are not always achievable. Numerous experiments are required with a univariate analysis method when there exist more than four variables in the design of an experiment (DoE). Therefore, an experimental design that can quickly and efficiently screen and identify for discontinuous or non-numeric variables would be very useful. To date, minimal research has been published on how to integrate multi-dimensional risk variable patterns in a DoE and how high-risk variables may impact the operation of UE. The L18-Hunter experimental design allows rapid design of experimental protocols with discontinuous or non-numeric variables, and experimental models of critical variables can be fit by standard least squares and effect screening modes [16].

In addition, the response variables in most experimental designs are focused on the extraction yield or the amount of a single component. However, the extract obtained is generally a multi-component matrix and the single component is only one of the indicators of the comprehensive effect [17]. Therefore, the biological activity of the entire extract cannot be well characterized by determining the attributes or content of a single component, since biological activity is often the effect of the synergistic action of multiple components in the extract. On the other hand, few studies have used biological indicators as response variables, such as antioxidant activity and antibacterial activity. Taking the biological activity of the extract as the response variables will help to screen out the extraction process variables of high biological activity, and the highly biologically active extract can be directly used as raw materials for food additives or medicines. For example, Assam tea has good antioxidant activity when extracted by ethanol and can be directly used in breakfast cereal food additives after being dried [18]. Another study showed that hibiscus methanol extract xanthine has oxidase activity inhibition so it can be directly used as an active pharmaceutical ingredient (API) for antidepressants [19].

In the present study, a new experimental design method, namely the L18-Hunter experimental design, was used to investigate the effects of extraction power, extraction time and temperature, extraction solvent and material size on the extracting of *Forsythia suspensa* seed oil. It is also worth mentioning that in addition to the yield of the extract, the biological activity index of antioxidant activity was also used as a response. To the best of our knowledge, the relevant design method has not been used to study the UE of *Forsythia suspensa* seed oil and is not even used in the extraction of other botanical materials. In addition, the results of UE were further compared with data of a conventional extraction method (reflux extraction) and an advanced extraction method (supercritical fluid extraction). This study helped to select suitable extraction methods for extracting *Forsythia suspensa* seed oil and provide a quick screening method for identifying high-risk variables in the process.

## 2. Results and Discussion 

### 2.1. Model Fitting of UE

#### 2.1.1. Analysis of the Model

UE has gained increasing attention because of its efficient energetics and reduced extraction time. Compared to the method of reflux with solvent, the UE method has unique advantages in extracting oil components, especially for the extraction of low molecular weight compounds from plant materials. It has been considered to be more effective in extracting phytochemicals from plant sources and easily adapted to the pilot and industrial scale [5]. Numerous studies have adopted orthogonal and response surface methodologies to analyze and optimize the UE process. In these experiments, the effects of extraction time, temperature and power on extraction efficiency were usually examined by the DoE [5]. However, due to the high number of variables affecting the UE process, and some variables being discontinuous and non-numerical types, it is often not an easy task to adopt the response surface methodology or orthogonal when there exists a large number of discontinuous numeric and non-numerical variables in the experimental design. Therefore, this study attempted to carry out an advanced L18-Hunter experimental design, which was suitable for multi-factor and non-continuous variables, to explore the extraction process of the *Forsythia suspensa* seed in order to quickly identify the high-risk variables of UE.

According to the process designed by the experimental design, data of the response values were obtained (Table 1). It was evident from results that various process variables had significant differences in the extraction efficiency and biologic activity of the extracts. The global yield was calculated by weighing, while the antioxidant activity was determined by the ability to scavenge DPPH radicals (DPPH (%)). Based on these values, the response variables were generated. The global yield and antioxidant activity ranges obtained in the experiments were 0.4%~16.0% and 1%–65%, respectively.

The screening model was used to statistically analyze the experimental results and establish effective models (Table 2). The estimate was the coefficient estimate of the variables; the larger the value, the greater the influence on the dependent variables, and the direction of the value indicated whether the effect on the dependent variable would be positive or negative. The P-value was used as a tool to check the significance of each variable. The smaller the P-value, the more significant the corresponding coefficient [20]. Moreover, R^2^ and RMSE were calculated to check the model’s accuracy. A higher R^2^ and a lower RMSE indicated that the variation could be accepted and that the model was reliable [21].

The results of the statistical analysis imply that the regression model was highly significant and the prediction ability of extraction yield and DPPH scavenging activity was accurate with R^2^ of 0.7925 and 0.9160, respectively. Generally, R^2^ was greater than 0.75, indicating the regression model was a good fit [22]. The analyzed results (Table 2) also show that the values of RSME were small, further confirming that a linear correlation existed between the predicted values and the actual levels of the responses.

#### 2.1.2. Model Fitting of Extraction Yield

**Figure 1:**
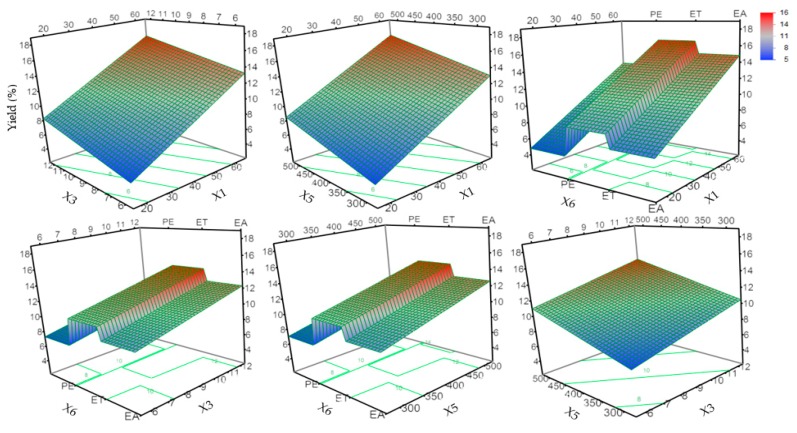
Graphical results in terms of the response of the yield from the developed model. Yield, extraction yield (%); X1, time (min); X3, solvent-to-solid ratio; X5, power (W); X6, type of solvent; ET, ethanol; EA, ethyl acetate; PE, petroleum ether.

The most effective way to visually observe the effect of independent variables on a response variable was to characterize the surface response plot of the model [23]. The effect of the variables on the extraction yield of the extract was determined by the model screening (Table 2). The response surface and contour plot in Figure 1 illustrate the significant variables (X1, X3, X5, X6) affecting the yield of oil. After neglecting the insignificant variables, the global yield can be described by the following model (Equation (1)):

(1) $$\mathrm{Yield}\;(\%)\;=\;10.64+3.26\times \left(\frac{X1-40}{20}\right)+1.37\times \left(\frac{X3-9}{3}\right)+1.46\times \left(\frac{X5-400}{100}\right)+Match\;\left(X6\right)\left(\begin{array}{c} EA\overset{}{\Rightarrow }0.09\\ ET\overset{}{\Rightarrow }1.68\\ PE\overset{}{\Rightarrow }-1.77 \end{array}\right)$$

where X1, X3 X5 and X6 are the coefficient of independent variables, extraction time, solvent-to-solid ratio and ultrasonic power and type of solvent, and match is the coefficient values corresponding to the different extraction solvent. 

This finding was in agreement with previous studies assessing the effectiveness of UE. Safaryan et al. reported that ultrasonic time and power significantly affected the yield of polysaccharides from green pea pods [4]. Sicaire et al. reported a similar influence of ultrasonic power in the extraction of oil from oleaginous seeds [15]. The general result of higher power in these studies led to higher global yields. A possible reason was that a higher system energy increased the solubility, thereby increasing release from the sample matrix by destroying the integrity of connective tissue and structural tissue.

Extraction time significantly affected the extraction efficiency (*p* = 0.0001). The purpose of extending the extraction time was to accumulate an increase in the energy applied to the sample matrix. Thus, increasing the energy of the system promoted the dissolution and release of the ingredient. However, it was not with the infinite extension of the extraction time that the yield was more effective. When a dissolution platform of the sample matrix was reached, the increase in the extraction time had no significant effect on the dissolution of compounds and thus did not change the extraction efficiency. Unfortunately, an excessive extraction time could lead to hydrolyzation or degradation [24]. Similar findings were reported in previous studies with regard to the extraction of polysaccharides from a Chuanxiong rhizome. In the study of extracting polysaccharides from a Chuanxiong rhizome by Hu et al., the extraction yield obviously increased when the ultrasonic time increase from 5 to 30 min, and then the yield reached a plateau period [14].

The solvent-to-solid ratio was one of the most important variables from an economical point of view. The yield increased with the increasing of power and the solvent-to-solid ratio. Generally, a large solvent-to-solid ratio and ultrasonic power could dissolve target compounds more effectively. It can be seen that the extraction yield increased with the increase of ultrasonic power from 300 to 500 W, and the solvent-to-solid ratio from 6 to 12.

The effect of the solvent type on the extraction efficiency was mainly due to the difference in the polarity of the solvent that caused the difference in dissolution ability. The extraction solvent ethanol and ethyl acetate had greater polarity than that of petroleum ether and therefore, showed a greater ability for dissolution, especially for polar substances. Additionally, ethanol and ethyl acetate were sometimes preferred for extraction due to their environmentally friendly nature.

#### 2.1.3. Model Fitting of DPPH Scavenging Activity

Plant seed oil contains many bioactive ingredients, which usually have antioxidant and anti-microbial effects [25]. The antioxidant ability of bioactive ingredients is mainly characterized by the activity of scavenging free radicals. Environmental pollution, ultraviolet radiation, smoking, etc., increase the amount of free radicals in the human body. Once the production of oxygen free radicals is excessive, or the ability to scavenge is weak, this causes cell damage and oxidative stress. At the same time, modern food processing technology has led to a decline in the nutritional function of food, increasing the demand for exogenous antioxidants. Looking for effective exogenous antioxidants is very beneficial for the treatment of diseases and human health. Modern pharmacological studies have confirmed that various plant components have antioxidant capacities. Therefore, the extraction of related components from plant oils as natural antioxidants is an effective research idea for plant development and utilization. Motivated by this need, the oils were subjected to screening for their potential antioxidant activity.

The developed prediction model showed good predictability of R^2^ = 0.9160 and RMSE = 7.9503 (Table 3). The first order model equation to predict the DPPH scavenging activity is given below (Equation (2)):

(2) $$\mathrm{DPPH}\;(\%)\;=\;27.43+\mathrm{Match}\;(X6)\left(\begin{array}{c} EA\overset{}{\Rightarrow }-5.79\\ ET\overset{}{\Rightarrow }25.06\\ PE\overset{}{\Rightarrow }-19.28 \end{array}\right)$$

where X6 is the coefficient of independent variables and type of solvent, and match is the coefficient values corresponding to the different extraction solvent.

**Figure 2:**
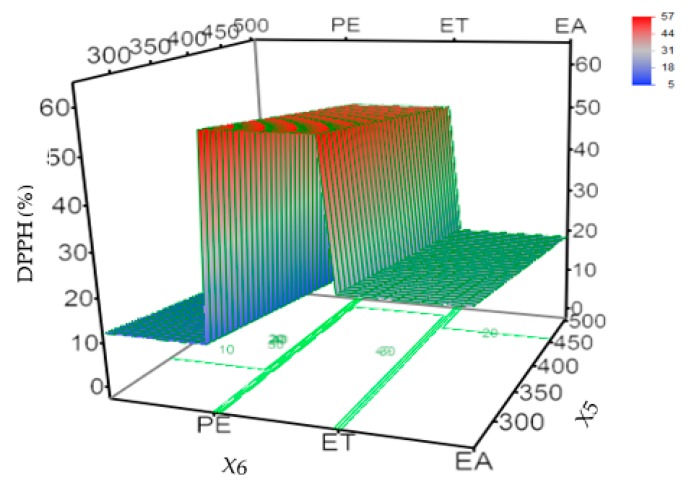
Graphical results in terms of the response of the scavenging activity of DPPH from the developed model. DPPH (%), DPPH scavenging activity (%); X5, power (W); X6, type of solvent; ET, ethanol; EA, ethyl acetate; PE, petroleum ether.

The results of regression analysis show that the type of solvent had a significant effect on antioxidant activity (Figure 2). The oil extracted by ethanol had strong antioxidant activity, while the oil extracted by non-polar components such as petroleum ether had weak antioxidant activity. Several studies have demonstrated that the antioxidant activity of Forsythia was mainly caused by compounds such as forsythin and forsythiaside A [3]. The IC_50_ (half maximal inhibitory concentration) of forsythiaside A was 0.43 μg/mL, exhibiting high activity. Ethanol can effectively extract forsythin and forsythiaside A, so the oil extracted by ethanol has high antioxidant activity. This result was also confirmed by the study by Wang et al. [2]. Moreover, the GC-MS results in Section 2.2 show that non-polar solvents, such as petroleum ether and ethyl acetate, extracted more weakly polar compounds of beta-Pinene and sabinene. These components exhibited good antibacterial activity rather than an antioxidant effect [26].

#### 2.1.4. Validation the Model

In order to assess the accuracy and predictability of the obtained models, the oil samples were prepared under three different process conditions. The difference between T-1, T-2 and T-3 was the extraction solvent used. Table 3 lists the difference between the experimental and predicted values of the response variables and was characterized by a percentage error (PE) [27]. It can be speculated from the results that the model had a strong predictive ability for the response variables and exhibited small PE values. It is worth mentioning that, although the model prediction ability was relatively high, the prediction ability deviation from the oil yield was 16.11% in T-3. The probable reason was that the R^2^ = 0.7925 for the yield model, and the regression yield model was less accurate than that of antioxidant model (DPPH R^2^ = 0.9160). By contrast, for the antioxidant model, the prediction ability of the model was more accurate when ethanol was used as a solvent (T-1). On the other hand, in the case of low antioxidants, when using petroleum ether, the prediction ability was poor. The change in the ultraviolet absorbance value was very small, and consequently the measurement fluctuation was large due to the operation or instrument error resulting in relatively large PE values. Therefore, the prediction ability for DPPH (%) was relatively weak. Nevertheless, both the prediction ability and accuracy of these fit models were within the allowable limits and acceptable.

The L18-hunter experimental design can be used to quickly screen the multivariate influencing variables and, especially in the case of non-continuous variables, the experimental method has obvious advantages [28,29]. Based on the comprehensive statistical analysis, it was confirmed that the critical process variables affecting the UE of *Forsythia suspensa* seed oil were type of solvent, solvent-to solid ratio, extraction power and time. Among these critical variables, the type of solvent was the most critical variable, and it had the greatest influence on the composition and antioxidant activity of the oil. These findings suggest that we could adopt a solvent-polarity-selection strategy to achieve extracts with the desired biological activity. At the same time, this study also investigated the effects of extraction temperature and raw material particle size on extraction efficiency. Changes in the above variables caused deviations in extraction results. However, under certain conditions, the impact of these variables on the extraction results was negligible and could be ignored.

### 2.2. Compared with Different Extraction Methods

Considering the current conventional methods of extraction seed oil, we investigated the differences in the physicochemical properties of the oils that were extracted by UE, SE, SFE and HD. The yields of *Forsythia suspensa* seed oil obtained by SE, SFE and HD were 16.48%, 8.57% and 8.01%, respectively. In addition, the oil extracted by different methods was characterized and compared by FT-IR, GC-MS and antioxidant activity. The yield of the oil obtained by SFE was lower than that reported in the literature. It has been reported that the yield of oil in artichokes obtained by SFE was close to the yield of oil collected by the SE [30]. Reports in the literature usually used a small amount of material, generally less than 10 g, extracted in a small instrument. The reason for the inconsistent oil yield by these two methods could be attributed to differences in the scale of the instrument. In this study, the size of the extraction vessel (2 L) and the collection vessel (500 mL) in the supercritical carbon dioxide system were large, and a part of the oil adhered to the surface of the extractor’s inner wall, failing to collect effectively. Additionally, it is also necessary to take into account the difference in oil and species of plants.

#### 2.2.1. FT-IR Spectroscopy Analysis

**Figure 3:**
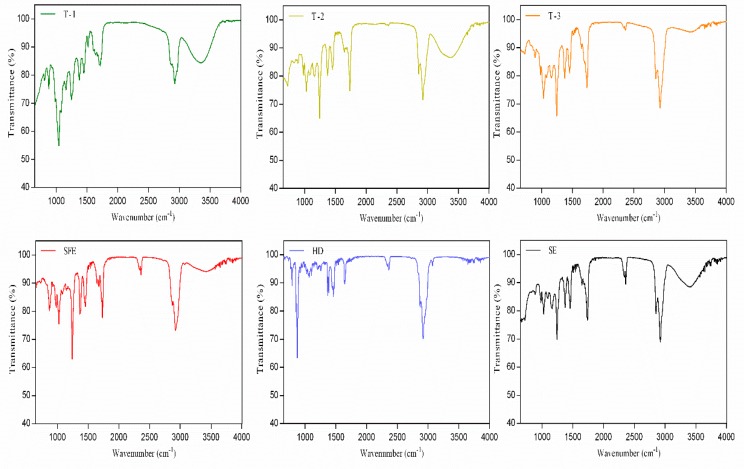
FT-IR spectra of oils extracted by methods T-1, T-2 and T-3, test formulation for ultrasonic extraction; SFE, supercritical fluid extraction; HD, hydrodistillation extraction; SE, Soxhlet extraction.

An FT-IR analysis was carried out to explore any differences that might exist in the extracted oils (Figure 3). FT-IR spectroscopy was used for approximate identification of the structure when combined with chemical analyses. The extracted oils were a complex mixture system, and their FT-IR spectra showed a total overlap of each absorption spectrum of various components [31].

It can be seen that the positions of the absorption peaks of the *Forsythia suspensa* seed oils prepared by different extraction processes were consistent in the infrared spectrum, suggesting that the extracts obtained by different extraction processes contained the same principal components or chemical functional groups. However, the intensity of the absorption peak was different, which may be attributable to a difference in the content of the ingredients contained in the extracts.

In the infrared spectrum, the method mainly reflected the absorption difference of 700–1300 cm^−1^ bands. For example, T-1 had a strong absorption peak around 1000 cm^−1^, while HD had a strong absorption near 900 cm^−1^ but exhibited weak absorption near 1250 cm^−1^. At a wavelength of 2921 cm^−1^, it was a C–H stretching vibration of methylene group, 2970 cm^−1^ for =CH_2_ symmetrical stretching vibration, 1642 cm^−1^ for H–O–H bending, 3400 cm^−1^ for O–H stretching vibration, 1083 cm^−1^ and 1033 cm^−1^ for symmetric C–O–C stretching vibration [32].

#### 2.2.2. Composition of Oil Obtained by Different Methods

Table 4 shows the list of chemical components of oil obtained by different methods and expressed as percentages. Different extraction processes or solvent polarity may affect the extract composition or change the extraction kinetics [33]. The principal components extracted by UE, SFE, SE and HD extraction methods were beta-Pinene, alpha-Pinene and sabinene. Monoterpene hydrocarbons and oxygenated monoterpenes were identified to be the major compounds in the extracted oils, which was consistent with the literature results with regard to the composition of *Forsythia suspensa* oil [34]. The components extracted by ultrasonic extraction (T-1) were more complex, and there were 18 components in the extract with a ratio of more than 1.0%. However, the content of beta-Pinene was dramatically lower (20.7%), while the content of Terpinen-4-ol was relatively high (22.6%). A higher concentration of oxygenated monoterpenes of Terpinen-4-ol was present in the oil isolated by UE (T-1). The reason for these results can be attributed to the greater polarity of the solvent used in ethanol extraction. Beta-Pinene, a monoterpene hydrocarbon, was the main abundant component in the oils for the UE (T-2, T-3), SFE, SE and HD methods: 46%~52.4%. Among these extraction processes, the extract obtained by SFE had the highest ratio of beta-Pinene, 52.4%, indicating that the non-polar carbon dioxide had higher solubility for non-polar beta-Pinene. Thus, a sufficient amount of beta-Pinene could be extracted using SFE. The main components of the oil extracted by a weakly polar solvent were beta-Pinene and sabinene. The compositions of oils obtained by different processes were relatively comparable when the polarities of the solvents were close. The results of the FT-IR absorption spectrum also confirm this view. Comparable results were obtained by Sodeifian et al. using supercritical fluid extraction and Soxhlet reflux extraction methods and the effect of the non-polar solvents on the chemical composition of the oil was negligible [35]. It was further confirmed that the polarity of a solvent is an important variable affecting the chemical composition of the extracted oil.

#### 2.2.3. Antioxidant Activity of Oil Obtained by Different Methods

**Figure 4:**
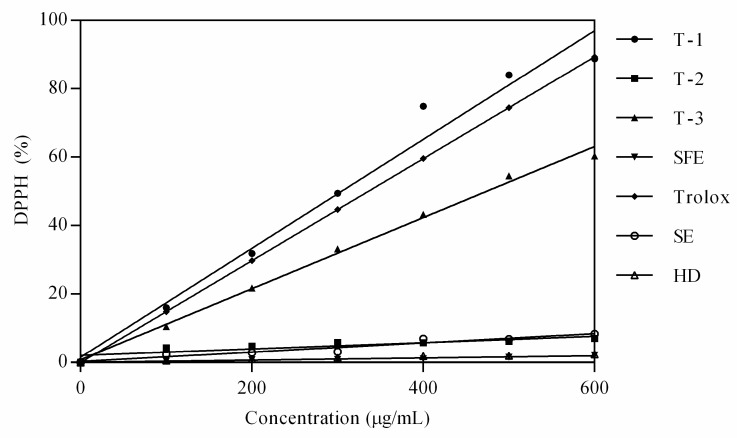
DPPH scavenging activity of oils extracted by different methods. T-1, T-2, T-3, test formulation for ultrasonic extraction; SFE, supercritical fluid extraction; HD, hydrodistillation extraction; SE, Soxhlet extraction.

Antioxidants have been extensively studied in food, agriculture and pharmaceutical industries, and DPPH assay has been widely used to determine the ability of samples to scavenge free radicals due to its simplicity and sensitivity [36]. We characterized the antioxidant activity of oil obtained by different extraction methods (Figure 4). Figure 4 shows that the DPPH scavenging abilities of oils were quite different. Among the oils extracted by these methods, oil extracted by ethanol had the highest antioxidant activity (T-1), showing intense free radical inhibition in the concentration range of 0~600 μg/mL. Followed by ethanol extract, oil extracted by ethyl acetate (T-3) also had some antioxidant activity, while the other oils had weak activity. It is worth mentioning that the antioxidant activity of ethanol-extracted oil was higher than that of Trolox. Data were calculated from the study of Thoo et al. [37]. Moreover, ethanol is a green environmentally friendly extraction solvent. Therefore, *Forsythia suspensa* seed oil may be a promising antioxidant dietary supplementation developed as a “natural” antioxidant to be used as an alternative to “synthetic” antioxidants in functional food and medicine via complementary extraction with ethanol. The possible mechanism by which oils acted as antioxidants may be attributed to their electron donation power to the free radicals, terminating the radical chain reaction [20]. According to the chemical composition analysis, 18 kinds of compounds (CMC ≥ 1.0) existed in the T-1 extract. It is speculated that the increase in the antioxidant activity of T-1 was linked to the synergy between multi-component and polar substances. Supplementary studies on compound purification, structure confirmation and in vivo antioxidant evaluation are in progress.

## 3. Materials and Methods

### 3.1. Samples and Reagents

Unbroken *Forsythia suspensa* seeds from Jin City of Shanxi province were supplied by Beijing Tong Ren Tang Chinese Medicine Co. LTD. The seeds were chopped in an RS-FS1401 universal grinder (Royalstar, Hefei, China). In order to avoid excessive pulverization, the grind time was fixed to 5 s and then sieved in time, and the operation was repeated until all the seeds were passed through the specific sieves. The mean diameter, for convenience, was assigned to a fraction based on sieve openings [38]. The powdered seeds were collected in three ranges of size 2, 3 and 4 mm. All the other chemical reagents used in the study were of analytical or HPLC grade.

### 3.2. Extraction of Oil

#### 3.2.1. L18 Hunter Design-Assisted UE

The UE was carried out in a KQ-500DE ultrasonic device (Kunshan ultrasonic instrument CO. LTD, Kunshan, China) with an adjustable ultrasonic power with a maximum of 500 W and frequencies of 40 kHz, equipped with a digital timer and a temperature controller [24]. The powder of crushed dry seeds (about 30 g) was accurately weighed and placed in an Erlenmeyer, then mixed with an appropriated amount of extraction solvent. The independent variables selected were extraction time (X1), temperature (X2), solvent-to-solid ratio (*v*/*w*, X3), particle size (X4), extraction power (X5) and type of solvent (X6). Of these six independent variables, X1–X5 were of a numerical nature, while X6 was a categorical variable for ethanol (ET), ethyl acetate (EA) and petroleum ether (PE) (Table 5). Three levels were investigated for all the variables. The Hunter L18 screening design was constructed using JMP-SAS Discovery 10 (SAS, Cary, NC, USA) in this study. According to the experimental design, materials were treated at the different operation conditions as described in Table 1. The responses measured were global yield and the DPPH scavenging activity of the extracted oils. The selection criteria and setting range of the independent variables were based on prior knowledge and literature reports. The response values considered were the global yield and biological activity of the extract. After extraction, the extraction solutions were centrifuged at 5000 rpm for 10 min at 4 °C to remove insolubles, then solvents were removed by a Eyela N-1300 rotary evaporator (Rikakikai Co., LTD, Tokyo, Japan) under vacuum at 40 °C. The amount of oil extracted was then calculated gravimetrically and stored at 4 °C in a refrigerator until analyzed.

#### 3.2.2. Soxhlet Extraction (SE)

For conventional organic compounds from solid samples’ extraction procedures, extraction carried out in a Soxhlet cartridge is the most common method [39]. Twelve grams of ground seeds (3 mm) was loaded in the glass thimble and maintained at reflux in a 250 mL Soxhlet apparatus; 180 mL PE was used as the extraction solvent (solvent-to-solid ratio: 15:1, *v*/*w*). Heating was stopped once the color of the condensed solvent at the top of the apparatus was clear and time was not less than 6 h. After extraction, the extraction solutions were centrifuged at 5000 rpm for 10 min at 4 °C to remove insolubles, and then solvents were removed by evaporation under vacuum at 40 °C. The amount of extracted oil was then calculated gravimetrically and stored at 4 °C in a refrigerator until analyzed.

#### 3.2.3. Hydrodistillation Extraction (HD)

For normal HD extraction in a Clevenger-type apparatus, 200 g of ground sample (3 mm) was immersed into 1400 mL water and distilled for up to 6 h. The essential oil was collected and the residue water was removed by adding 0.5 g of anhydrous sodium sulfate, then weighed [40]. The oil was stored at 4 °C in a refrigerator until analyzed.

#### 3.2.4. Supercritical Fluid Extraction (SFE)

Supercritical carbon dioxide extraction was carried out using a laboratory-scale supercritical fluid Waters Bio-Botanical extraction system SFE-500M1-2-C50 (Waters, Milford, MA, USA) [41]. The system consisted of a high-pressure CO_2_ pump (maximum pressure = 600 bar) to pressurize the liquefied CO_2_ to the desired pressure, a 2-L extraction vessel surrounded by a heating jacket, and a 500-mL collection vessel. Moreover, 120 g of *Forsythia suspensa* seed powders with a particle size of 3 mm were manually packed into the stainless steel extractor. The CO_2_ flow rate, pressure and temperature of the extraction vessel modulated by ChromScope Process Suite software v5.9 were controlled at 40 g/mL, 350 bar and 45 °C, respectively. Each extraction lasted 120 min. In order to avoid the extracted oil being heated and volatilized, the temperature and pressure of the collection vessel were maintained at 10 bar and 0 °C, respectively. The SFE process parameters selected were based on previous data obtained in our laboratory.

### 3.3. Determination of Extraction Yield (Yield %)

The global yield from all the extracts (Equation (3)) was calculated as follows:

(3) $$\mathrm{Yield}\;(\%)\;=\;\frac{M_{oil}}{M_{raw}}\times 100\%$$

where M_oil_ is the mass fraction of extracted oil and M_raw_ is the mass fraction of raw material subjected to extraction.

### 3.4. Characterization of Oil by GC-MS

Gas chromatography and mass spectrometry (GC-MS) (Shimadzu QP 2010 Plus, Shimadzu, Kyoto, Japan) equipped with National Institute of Standards and Technology (NIST) v14 (Department of Commerce, Washington, USA) and Flavor and Fragrance Natural and Synthetic Compounds (FFNSC) v1.2 (Shimadzu, Kyoto, Japan) as standard libraries were utilized for the analysis of the oil extracted. The extract oil was dissolved in HPLC grade methanol prior to the injection with concentration of 8 mg/mL. In each analysis, about 1.0 μL of a diluted sample was injected into the GC/MS system equipped with a mass selective detector. The operating conditions were the following: Capillary column Agilent J&W VF-WAXms (30 m × 250 μm × 0.25 mm); helium as carrier gas: 1.0 mL/min; injector temperature: 220 °C; capillary column temperature programmed at 50 °C isothermal for 3 min, and then increased to 150 °C at a rate of 5 °C/min and held isothermal for 2 min, and increased to 220 °C at a rate of 10 °C/min isothermal for 4 min, and final increase to 230 °C at a rate of 40 °C/min and held for 2 min; 1:5 the split ratio; ionization voltage: 70 eV; ion source temperature: 200 °C; mass scan ranges: 30–600 mass units, scan rate of 5000 cycles per second.

The identification of major compounds was based on the comparison of retention induced on the column with literature data and computer matching those mass spectra fragmentation patterns stored in FFNSC v1.2 and NIST v14. The composition of major compounds (CMC) was calculated from the total ion chromatograms by an integrator, as shown in Equation (4) [42];

(4) $$\mathrm{CMC}\;(\%)\;=\;\frac{A_{m}}{A_{t}}\times 100\%$$

where A_m_ and A_t_ are the peak area of major compounds and the total peak area of major compounds.

### 3.5. Determination of Antioxidant Activity: DPPH Assay (DPPH %)

The effect of scavenging the DPPH (2, 2-diphenyl-1-picryhydrazyl) radical activity was determined as previously described by Deng et al. and Benito-Román et al. [43,44] with minor modifications. Briefly, 0.4 mL of sample ethanol solution (300 μg/ml) was mixed with 2 mL of 0.1 mM DPPH radical ethanol solution. The mixture was vigorously shaken for 1 min in a vortex mixer. The reaction mixture was left to incubate at room temperature for 30 min in the dark and the absorbance was measured at 517 nm using UV-vis spectrophotometry (SPECORD® 50 PLUS). The scavenging activity of DPPH (%) was calculated as follows (Equation (5)):

(5) $$\mathrm{DPPH}\;(\%)\;=\;(1\;-\;\frac{A_{sample}}{\;A_{control}})\times 100\%$$

where A_sample_ is the absorbance of the test sample and A_control_ is the absorbance of the negative control which was measured by replacing the sample with 0.4 mL of ethanol.

### 3.6. FT-IR Spectroscopy

Fourier transform infrared (FT-IR) spectroscopy was performed using a Nicolet iS 50 spectrometer (Thermo Scientific^TM^, Waltham, MA, USA) coupled with a Smart iTR diamond ATR^TM^ accessory, and absorption spectra were recorded at wavelengths of 4000 to 400 cm^−1^. A drop liquid sample material was placed and spread evenly on the diamond window of the ATR^TM^ accessory and the sample was directly analyzed using Smart iTR. The resolution was optimized to 4 cm^−1^ and 16 scans respectively. A background spectrum obtained against air from the clean diamond ATR crystal was recorded and subtracted automatically by the OMNIC 9 software package (Thermo Scientific^TM^, Waltham, MA, USA) [45].

## 4. Conclusions

In this study, an L18-Hunter experimental design was utilized in UE to quickly screen the high-risk variables affecting the yield and antioxidant ability of *Forsythia suspensa* seed oil. Based on the results of the statistical analysis, type of solvent, solvent-to solid ratio, extraction power and time were high-risk variables, which were the process variables critical in determining the performance of UE. The type of solvent was the most critical variable in changing ultrasonic effects such as the antioxidant activity and oil composition of the extracts. The regression coefficients of the yield and antioxidant activity models were 0.79 and 0.91, and the ANOVA of the models were 0.013 and <0.0001, respectively, which showed a good correlation with the values predicted by the models. The L18-Hunter design could be used as an effective experimental design method for quickly screening and identifying high-risk variables when in complex conditions of multivariate non-continuous variables.

Moreover, the effects of UE, SE, SFE and HD processes on the composition, structure and antioxidant activity were compared. The results indicate that UE with ethanol as the solvent (T-1) could improve oil quality and tended to extract higher antioxidant activity than the other methods. Due to its particularly high antioxidant activity, oil extracted by ethanol can be used as a good potential source for medicines and a health promoting source in diets. The compositions of the oils extracted with UE (T-2, T-3), SE, SFE and HD were not significantly different. The main extraction component of these methods was beta-Pinene and the content was 46%~52.4%. The overall findings indicate that UE had an extremely high extraction efficiency of *Forsythia suspensa* seed oil, but attention should be paid to the selection of a suitable polar solvent.

## Figures and Tables

**Table 1:** L18-Hunter design and observed responses of oils extracted from *Forsythia Suspensa* seeds using ultrasonic extraction.

Run	Pattern	X1	X2	X3	X4	X5	X6	Yield (%)	DPPH (%)
1	000001	40	45	9	3	400	ET	14.9	54.9
2	−−−−−1	20	30	6	2	300	PE	0.5	6.0
3	−.0−−1	20	60	6	2	500	ET	8.6	54.6
4	−−.6−1	20	30	12	4	300	EA	9.5	33.8
5	−7.6−1	20	60	6	4	300	PE	3.6	13.0
6	−−−−−1	20	30	6	2	300	PE	0.4	6.2
7	++−+−0	60	60	6	4	300	PE	11.9	22.9
8	++++++	60	60	12	4	500	PE	15.8	1.8
9	000003	40	45	9	3	400	PE	10.3	5.2
10	+−−++−	60	30	6	4	500	ET	15.4	48.7
11	−1.4+−	20	60	12	2	300	ET	10.5	45.7
12	+−−−−+	60	30	6	2	300	PE	9.0	1.3
13	+++−−−	60	60	12	2	300	ET	12.5	55.3
14	−−.3−−	20	30	12	2	500	PE	12.8	9.3
15	−5.8−−	20	60	12	4	500	PE	3.5	3.1
16	+−+−+0	60	30	12	2	500	PE	13.4	10.5
17	+−++−−	60	30	12	4	300	ET	15.2	64.5
18	−−−+−−	20	30	6	4	500	ET	9.2	43.7
19	000002	40	45	9	3	400	EA	9.0	12.2
20	++−−+0	60	60	6	2	500	PE	13.3	14.6
21	++++++	60	60	12	4	500	PE	15.1	1.3

X1, time (min); X2, temperature (°C); X3, solvent-to-solid ratio; X4, particle size (mm); X5, power (W); X6, type of solvent; ET, ethanol; EA, ethyl acetate; PE, petroleum ether; Yield, extraction yield (%); DPPH (%), DPPH scavenging activity (%).

**Table 2:** ANOVA analysis for the screening model developed for ultrasonic extraction.

Term	Yield (%)		DPPH (%)	
	Estimate	*p*-Value	Estimate	*p*-Value
Intercept	10.64	<0.0001	27.43	<0.0001 ^*^
X1	3.26	0.0003 ^*^	1.23	0.5380
X2	−0.07	0.9119	0.17	0.9299
X3	1.37	0.0592 ^*^	0.10	0.9597
X4	0.24	0.7271	1.04	0.6051
X5	1.46	0.0439 ^*^	−2.94	0.1557
X6 [EA]	0.09	0.9498	−5.79	0.2041
X6 [ET]	1.68	0.1114	25.06	<0.0001 ^*^
X6 [PE]	−1.77	0.0853 ^*^	−19.28	<0.0001 ^*^
ANOVA		0.0013 ^*^		<0.0001 ^*^
R^2^	0.7925		0.9160	
RMSE	2.6787		7.9503	

^*^p-Value < 0.10; Yield, extraction yield (%); DPPH (%), DPPH scavenging activity (%); X1, time (min); X2, temperature (°C); X3, solvent-to-solid ratio; X4, particle size (mm); X5, power (W); X6, type of solvent; ET, ethanol; EA, ethyl acetate; PE, petroleum ether; ANOVA, analysis of Variance; R^2^, coefficient of determination; RMSE, root mean square error.

**Table 3:** Experimental values and predicted values of response variables.

		Yield (%)	DPPH (%)
T–1	PV	17.1	50.8
	EV	17.0	49.4
	PE	−0.2	−2.8
T–2	PV	15.5	19.9
	EV	14.0	20.6
	PE	−10.1	3.1
T–3	PV	13.6	6.5
	EV	16.2	4.1
	PE	16.1	−56.2

T-1, T-2, T-3, test formulation for ultrasonic extraction; Yield (%), extraction yield (%); DPPH (%), DPPH scavenging activity (%); PV, predicted values; EV, experimental values; PE, percentage error, PE = 100 × (EV − PV)/EV.

**Table 4:** Contents of the identified compounds in the oils isolated from seeds by different methods ^a.^

No.	Compounds	CAS	Peak Area (%)					
			Ultrasonic Extraction			SFE	HD	SE
			T-1	T-2	T-3			
1	alpha-Pinene	80-56-8		10	10.6	13.3	20.4	15.9
2	alpha-Thujene	2867-5-2	1.2	1.1			1.1	
3	beta-Pinene	127-91-3	20.7	46.0	46.1	52.4	46.7	47.2
4	Sabinene	3387-41-5	11.4	20.9	18.8	19.4	17	18.6
5	Mycrene	123-35-3	2.4	3.2	2.8	2.5	2.4	2.6
6	alpha-Terpinene	99-86-5	1.0				1.0	
7	Limonene	138-86-3	2.3	2.4	2.1	2.1	1.8	2.1
8	Eucalyptol	470-82-6	4.2					
9	Terpinene	99-85-4	3.1	1.3				
10	gama-Terpinene	99-85-4			1.0		1.8	1.1
11	Cymene	99-87-6	1.4					
12	Pimelic ketone	108-94-1	1.6					
13	Menth-2-en-1-ol	29803-82-5	1.3					1.1
14	Sabinene hydrate	17699-16-0	7.3					1.1
15	trans-Sabinene hydrate	176699-16-0	8.7	1.3				
16	Terpinen-4-ol	562-74-3	22.6	4.3	2.8	1.4	3.5	3.8
17	trans-Pinocarveol	1674-08-4	1.0					
18	alpha-Terpineol	98-55-5	2.0					
19	gama-Cadinene	39029-41-9	1.4					
20	delta-Cadinene	483-76-1	2.0					

^a^ Compounds are listed with CMC ≥ 1.0. T-1, T-2, T-3, test formulation for ultrasonic extraction; SFE, supercritical fluid extraction; HD, hydrodistillation extraction; SE, Soxhlet extraction.

**Table 5:** Processing conditions and values for the variables in the design of experiments.

Independent Variables		Level in the Experiments			Test Formulation		
		−1	0	1	T-1	T-2	T-3
Time (min)	X1	20	40	60	60	60	60
Temperature (°C)	X2	30	45	60	45	45	45
Solvent-to-solid ratio	X3	6	9	12	12	12	12
Particle size (mm)	X4	2	3	4	3	3	3
Power (W)	X5	300	400	500	500	500	500
Type of solvent	X6	ET	EA	PE	ET	PE	EA

ET, ethanol; EA, ethyl acetate; PE, petroleum ether.

## Supplementary Materials

The Supplementary Materials are available online.
