# Supplementary material for: Quickly Identifying High-Risk Variables of Ultrasonic Extraction Oil from Multi-Dimensional Risk Variable Patterns and a Comparative Evaluation of Different Extraction Methods on the Quality of Forsythia suspensa Seed Oil
Source: Molecules. 2019 Sep 23;24(19):3445. doi: 10.3390/molecules24193445 (PMC6803820; doi:10.3390/molecules24193445)
Supplement: Supplementary file 1 [file molecules-24-03445-s001.zip › Supplementary data-final/Draft of Graphical Abstract .pptx]

## Slide 1
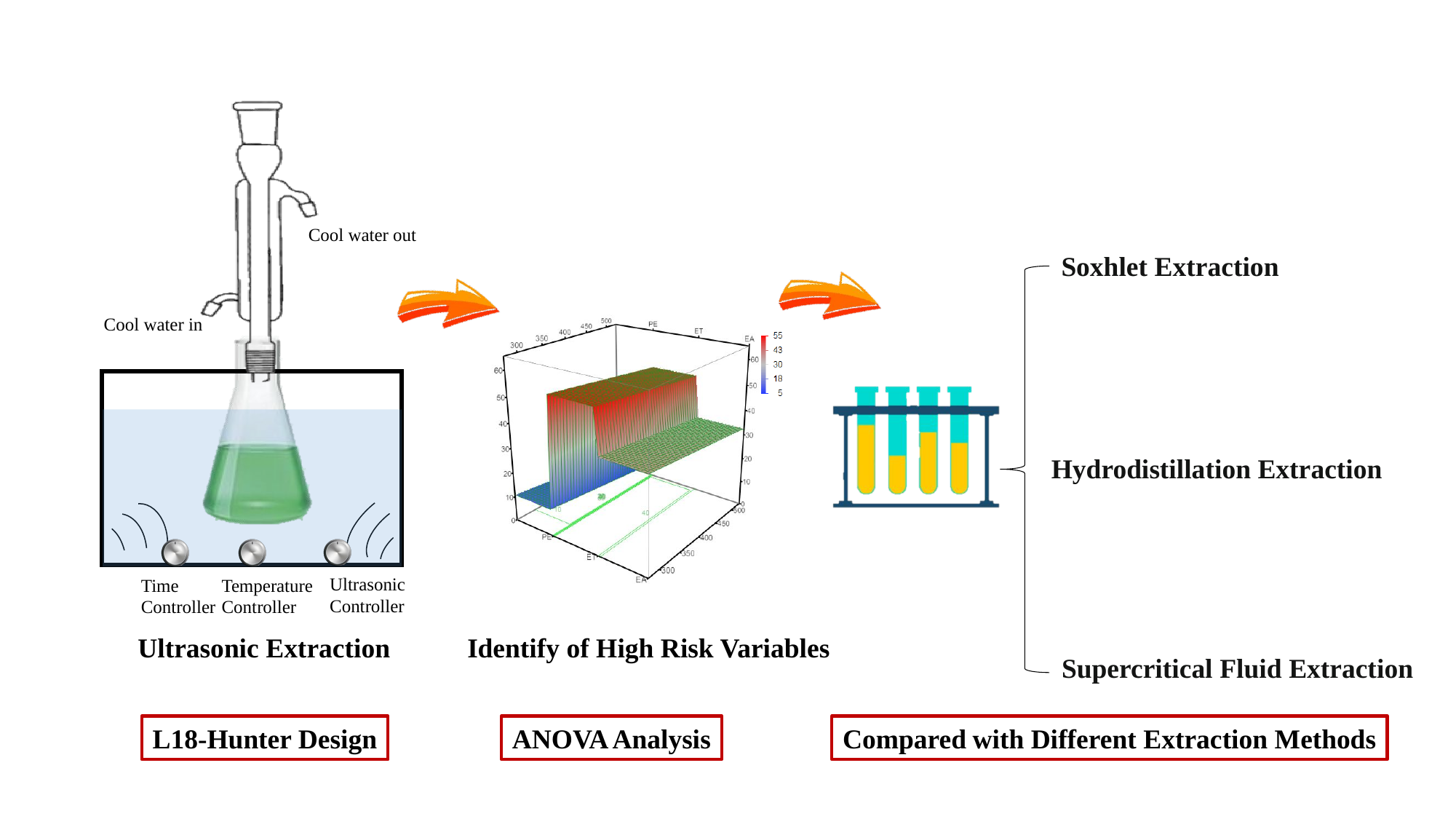

Cool water out
Soxhlet Extraction
Cool water in
Hydrodistillation Extraction
Ultrasonic
Controller
Time
Controller
Temperature
Controller
Ultrasonic Extraction
 Identify of High Risk Variables
Supercritical Fluid Extraction
L18-Hunter Design
ANOVA Analysis
Compared with Different Extraction Methods
