# Supplementary figures and images for: Quickly Identifying High-Risk Variables of Ultrasonic Extraction Oil from Multi-Dimensional Risk Variable Patterns and a Comparative Evaluation of Different Extraction Methods on the Quality of Forsythia suspensa Seed Oil
Source: Molecules. 2019 Sep 23;24(19):3445. doi: 10.3390/molecules24193445 (PMC6803820; doi:10.3390/molecules24193445)

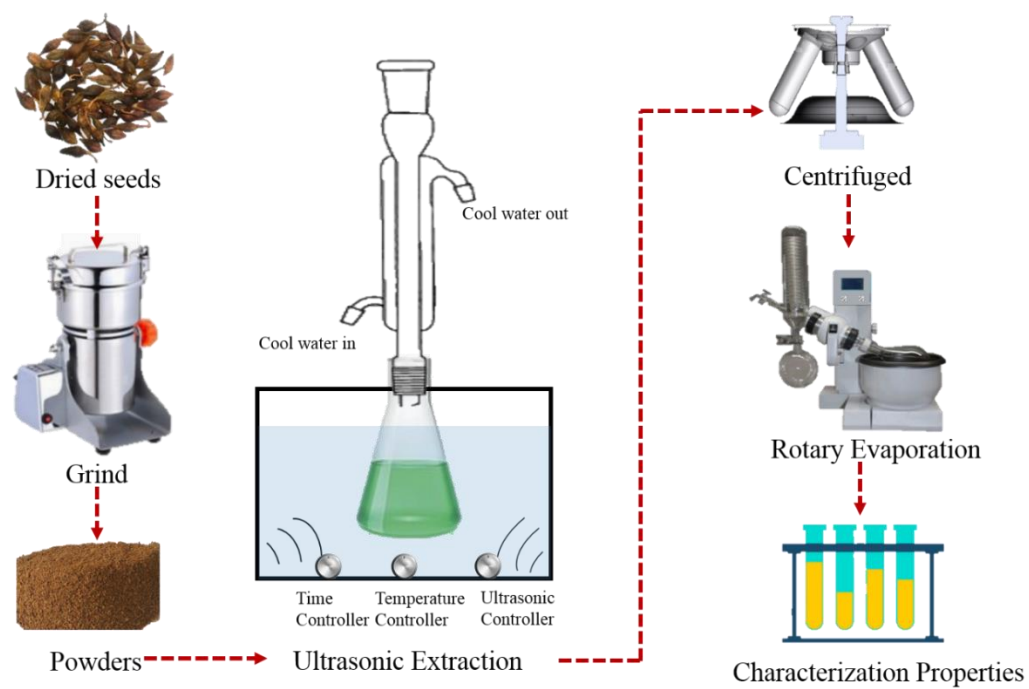

**Figure S1.** The schematic diagram of ultrasonic extraction protocol.

Supplement: Supplementary file 1 [file molecules-24-03445-s001.zip › Supplementary data-final/Supplement Material-Figure S1.pdf]

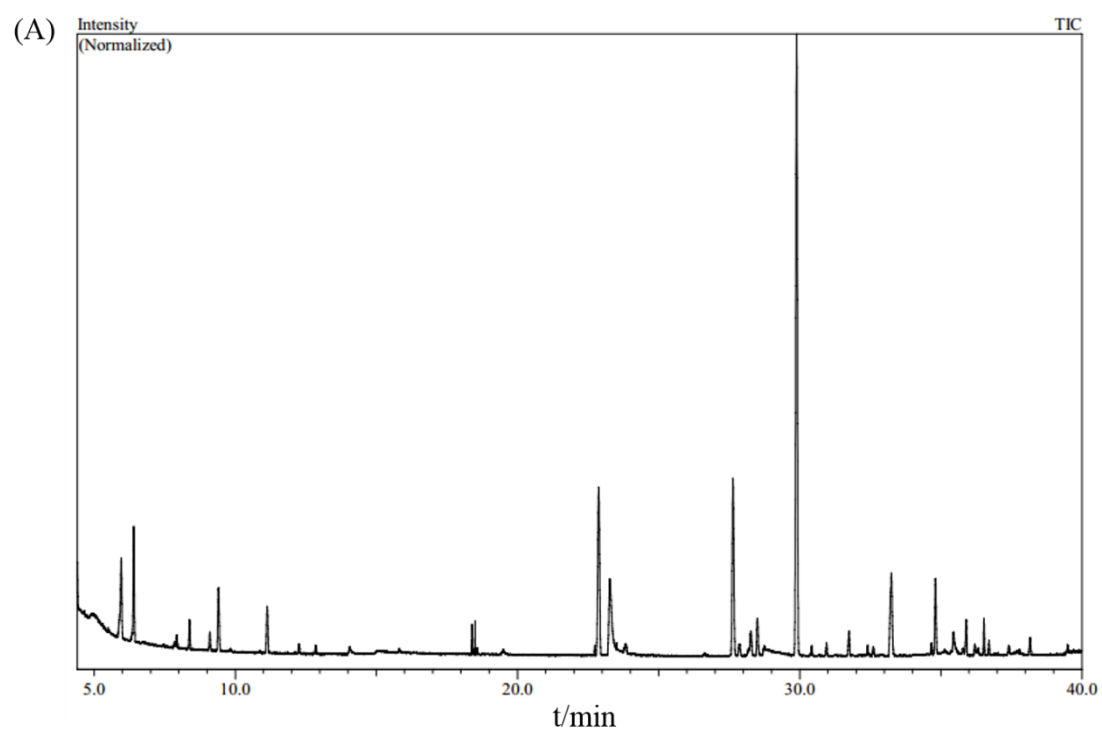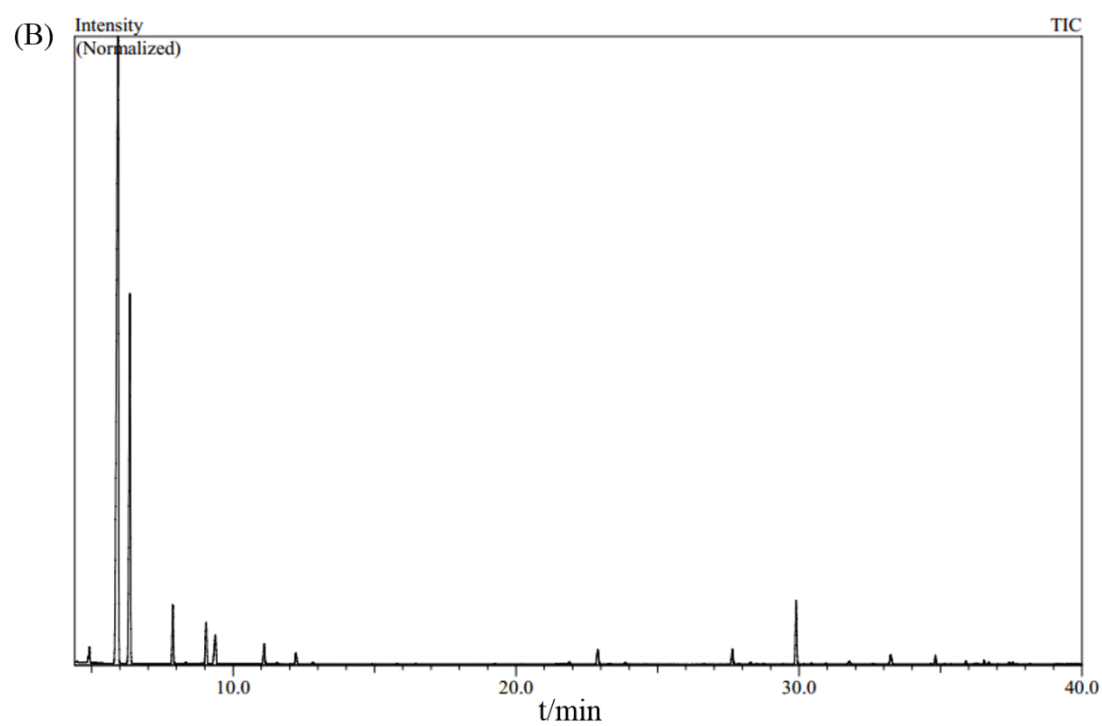

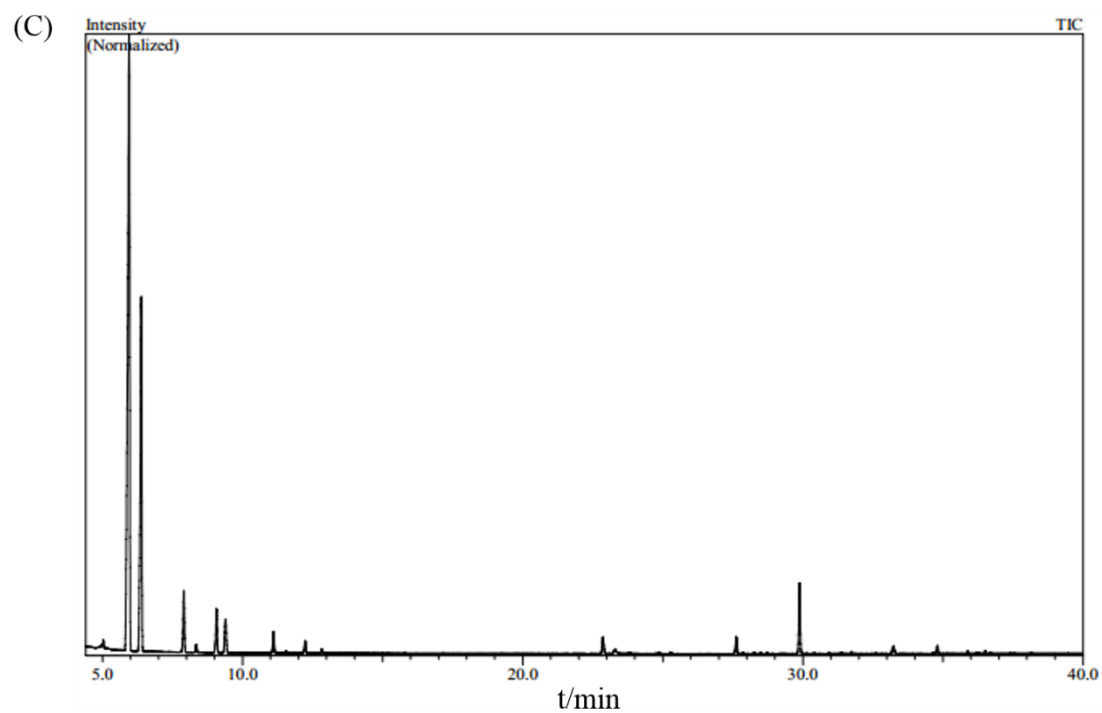

**Fig. S** Total ion chromatography (TIC) of oil extracted by ultrasound (A: Run 1, B: Run 9, C: Run 19)

Supplement: Supplementary file 1 [file molecules-24-03445-s001.zip › Supplementary data-final/Supplementary Figure-Total ion chromatography of oil extracted by ultrasonic extraction (Run #1#9#19).pdf]
